# Simultaneous Electrochemical Detection of LDL and MDA-LDL Using Antibody-Ferrocene or Anthraquinone Conjugates Coated Magnetic Beads

**DOI:** 10.3390/ijms24066005

**Published:** 2023-03-22

**Authors:** Daria Rudewicz-Kowalczyk, Iwona Grabowska

**Affiliations:** Institute of Animal Reproduction of Food Research, Polish Academy of Sciences, Tuwima 10, 10-748 Olsztyn, Poland

**Keywords:** low-density lipoprotein (LDL), malondialdehyde-modified low density lipoprotein (MDA-LDL), electrochemical detection, antibody-redox active agent conjugates, magnetic beads

## Abstract

The simultaneous detection of atherosclerotic cardiovascular disease (ACSVD) biomarkers was recently of great scientific interest. In this work, magnetic beads-based immunosensors for the simultaneous detection of low density lipoprotein (LDL) and malondialdehyde-modified low density lipoprotein (MDA-LDL) were presented. The approach proposed was based on the formation of two types of specific immunoconjugates consisting of monoclonal antibodies: anti-LDL or anti-MDA-LDL, together with redox active molecules: ferrocene and anthraquinone, respectively, coated on magnetic beads (MBs). The decrease in redox agent current in the concentration range: 0.001–1.0 ng/mL for LDL and 0.01–10.0 ng/mL for MDA-LDL, registered by square wave voltammetry (SWV), was observed upon the creation of complex between LDL or MDA-LDL and appropriate immunoconjugates. The detection limits of 0.2 ng/mL for LDL and 0.1 ng/mL for MDA-LDL were estimated. Moreover, the results of selectivity against the possible interferents were good, as human serum albumin (HSA) and high density lipoprotein (HDL), stability and recovery studies demonstrated the potential of platform proposed for early prognosis and diagnosis of ASCVD.

## 1. Introduction

According to the World Heart Federation, atherosclerosis cardiovascular disease (ASCVD) is a group of disorders that includes, among others, coronary heart disease (CHD), stroke, peripheral vascular disease (PVD) and myocardial infarction (MI). It is estimated that around 33% of all global deaths are caused by these diseases. The risk factors include the following: high blood pressure, high cholesterol, being overweight/obesity, air pollution, physical inactivity, unhealthy diet, diabetes, tobacco and harmful use of alcohol (https://world-heart-federation.org (accessed on 17 March 2023)).

Low density lipoprotein (LDL) and malondialdehyde-modified low density lipoprotein (MDA-LDA) are biochemical risk biomarkers that play an important role in the development of ASCVD. LDL is an example of a classical biomarker specific for patients with a high or very high cardiovascular risk [1]. Moreover, the oxidized lipids are classified as highly atherogenic components. Belonging to this group of lipids, MDA-LDL is an independent diagnostic and prognostic biomarker of atherosclerosis correlated with post infarct cardiosclerosis [2].

The LDL level of 100 mg/dL is estimated as normal value. However, LDL level of 190 mg/dL or higher raises the risk of cardiovascular disease up to very high [3]. In the case of MDA-LDL, it was demonstrated as having about a 7.2-fold higher plasma level of MDA-LDL for unstable atherosclerotic cardiovascular disease patients (1.3 mg/dL) as compared with control subjects [4]. Thus, regular monitoring of serum biomarkers is extremely important in diagnosis and monitoring of ASCVD different stages.

In routine clinical laboratories, Friedewald formula: LDL = TC-HDL-TGs/5 is used for estimation of LDL level. It takes into account concentration of total cholesterol (TC), high density lipoprotein (HDL) and triglycerides (TGs) [5]. However, this formula has some limitations and is only valid for samples with a TGs concentrations of less than 400 mg/dL, while enzyme-linked immunosorbent assay (ELISA) for the quantification of MDA-LDL in human plasma was developed and validated [6].

There is constantly growing research interest in development of new and advanced diagnostic tools for rapid, sensitive and accurate biomarkers detection in order to assess atherosclerotic cardiovascular disease prognosis and diagnosis [7]. Since ACSVD is a very large group of disorders, the detection of one biomarker is not enough for proper and timely diagnosis. One possible solution to this problem is the multiplexed detection of few biomarkers in a single analysis [8]. Among different detection techniques used in multiplexed approaches, electrochemical biosensors recently gained much scientific interest, including immunosensors [9] and aptasensors [10].

An extensive literature analysis showed that simultaneous electrochemical detection of LDL and MDA-LDL was not presented until now. However, several examples of electrochemical immunosensors for detection of LDL were published. Most of these biosensors need the presence of redox couple (e.g., ferricyanide/ferrocyanide ions (Fe(CN)_6_^3−/4−^)) in the supporting solution. The formation of immunocomplex between LDL and antibody leads to blocking the electron transfer between redox couple and electrode surface. Then, the redox signal changes depend linearly on LDL concentration [11,12,13,14,15,16,17]. In the other type of immunosensors, the redox label is either covalently and directly immobilized on the surface of electrode or covalently conjugated with receptor, e.g., an antibody. For example, NiO thin film supported antibody was used for the detection of LDL by the changes in the oxidation state of Ni (II/III) [18,19]. As a part of research in our group, we recently showed that the antibody ferrocene conjugates can act as an electrochemical platform for LDL detection [20]. In this case, upon the interfacial immunocomplex formation between antibody-ferrocene conjugates and LDL, the decrease in ferrocene redox current was registered. The changes in the redox current were correlated with concentration of LDL. The superior limit of detection was obtained as 0.53 ng/mL.

MDA-LDL is a key component of oxidized LDL (oxLDL). The electrochemical immunosensor for the detection of MDA-LDL was not presented. There are single examples of immunosensors for determination of oxLDL or electronegative LDL (LDL^−^). The electrochemical immunosensor based on adsorption of anti-LDL^-^ monoclonal antibody on a (polyvinyl formal)-gold nanoparticles modified gold electrode exhibited a sensitive response to LDL^−^, registered using electrochemical impedance spectroscopy and cyclic voltammetry [21]. The immunosensor based on immobilization of three monoclonal antibodies against oxLDL on self-assembling cysteamine with using EDC/NHS chemistry displayed a sensitive response from 0.5 to 18.0 µg/mL towards oxLDL [22].

The approach proposed in this paper involved applying an anti-LDL and an anti-MDA-LDL monoclonal antibodies co-immobilized with electroactive ferrocene or anthraquinone on magnetic beads (MB), respectively. Such platforms were used for simultaneous electrochemical sensing of LDL or MDA-LDL on the screen-printed electrodes (SPEs) using external magnetic field. LDL and MDA-LDL form specific immunocomplexes with appropriate antibodies-redox active agent conjugates-coated magnetic beads. In the presence of target biomarkers, the decrease in the redox current of electroactive ferrocene of anthraquinone was observed as a result of spatial blocking, registered by square wave voltammetry (SWV). This change is proportional to the concentration of corresponding analytes and is the base of simultaneous LDL and MDA-LDL detection.

SPEs and MB were appropriate for parallel LDL and MDA-LDL detection. Once the strategy was developed for LDL, it was adapted for MDA-LDL detection, except that the corresponding antibody and redox tag were changed.

## 2. Results and Discussion

### 2.1. Magnetic Dynabeads Conjugation with Antibodies and Ferrocene or Anthraquinone

Magnetic dynabeads recently became very popular, mainly for reasons such as biocompatibility, effective conjugation ability with the most of biological receptors and easy manipulation via magnetic separation technology [23]. Within this work, we applied magnetic dynabeads (Dynabeads^®^ M-270 amine, DB-NH_2_)-coated with amine groups as a solid support for antibodies. Surface-reactive primary amino-groups present on the beads allow for receptors immobilization through amide bond formation with carbodiimide—activated carboxylic groups from antibodies. We recently proved that linkers possessing NH_2_ groups are advantageous for covalent immobilization of antibody [17]. This phenomenon was confirmed by others as well [24,25,26].

**Scheme 1:**
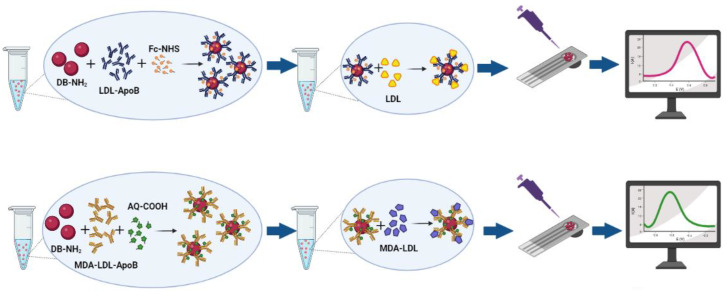
The procedure of magnetic dynabeads modification and the signal generation mechanism illustration.

In order to distinguish the beads by electrochemistry, they were additionally coated with redox active agents. Here, we prepared two kinds of magnetic dynabeads: (**1**) DB-NH_2_-coated with monoclonal antibody to apolipoprotein B specific for low density lipoprotein (LDL) (LDL-Apo-B) and an amine reactive sulfo-NHS ester (N-hydroxysulfosuccinimide) of ferrocene (Fc-NHS) and (**2**) DB-NH_2_-coated monoclonal antibody to malondialdehyde (MDA) modified LDL (MDA-LDL-Apo-B) and anthraquinone monocarboxylic acids (AQ-COOH) (Scheme 1).

Once coupled with specific antibodies and redox active molecules, DBs, upon deposition on the carbon screen-printed electrodes using a magnet, displayed the electrochemical signals observed as peak current at positive potential values for DB-LDL-ApoB-MAb-Fc and negative for DB-MDA-LDL-ApoB-Mab-AQ conjugates, registered using square wave-voltammetry (SWV). Afterward, the sample containing LDL or MDA-LDL target molecules were added to the beads and incubated. Upon affinity capture of the LDL or MDA-LDA by conjugated beads, the current was measured again and quantitative detection of targets was carried out. The schematic diagram of the coating procedure of magnetic dynabeads with respective antibodies and redox active agents together with signal generation mechanism is presented in Scheme 1. However, the details of coating procedure were described in the Materials and Methods section. In short, dynabeads-NH_2_ were used for capturing monoclonal antibodies anti-LDL-ApoB and anti-MDA-LDL-ApoB together with Fc-NHS and AQ-COOH (activated by EDC/NHS) in the first step, respectively. The dynabeads, thus modified, were ready for application in the simultaneous detection of LDL and MDA-LDL.

### 2.2. Optimization of Dynabeads Conjugation Procedure

The deposition of receptor on a solid support in an appropriately oriented manner is extremely essential in the process of immunosensor construction. In our previous papers, we proved the proper orientation of antibodies when deposited via NH_2_ linkers deposited on solid support [17,20]. According to the manufacturer description, the dynabeads M-270 amine were uniform and supermagnetic with a diameter of 2.7 µm. Their surface was coated with a hydrophilic layer of glycidyl ether activated with primary amine. Wherefore, the dynabeads were characterized by low non-specific binding, very good dispersion abilities and easy handling. We optimized the following parameters: the types of electrodes: carbon or gold, the types of buffer used for conjugation and measurements and the number of steps in conjugation (data not presented). Finally, we selected screen-printed electrodes made of carbon for further measurements, the measuring buffer with the following composition: 50 mM Tris, 0.15 M Na_2_SO_4_ pH 7.0 and one-step conjugation of DB with antibodies and redox agents together. The motivation was to obtain any additional peaks of unmodified DB when deposited on working electrode in a specified buffer and a nicely resolved peak current of Fc and AQ when modified with DB.

We selected two redox active markers for dynabeads conjugation: ferrocene (Fc) and anthraquinone (AQ). Ferrocene belongs to the family of organometallic sandwich complexes, and its chemical structure contains the central iron atom located between two cyclopentadienyl rings. This complex shows a higly reversible redox reactions according to the following equation: [Fe^III^(C_5_H_5_)_2_]^+^ +1 e^−^ → [Fe^II^(C_5_H_5_)_2_] [27,28]. Anthraquinone (AQ), an aromatic organic compound, reduced/oxidized according to the scheme: anthraquinone + 2 e^−^ +2H^+^ → anthrahydroquinone, is another alternative redox agent, since it is stable and easily attachable to biomolecules. Both agents were recently successfully adapted as redox agents in electrochemical immunosensors [20,29].

**Figure 1:**
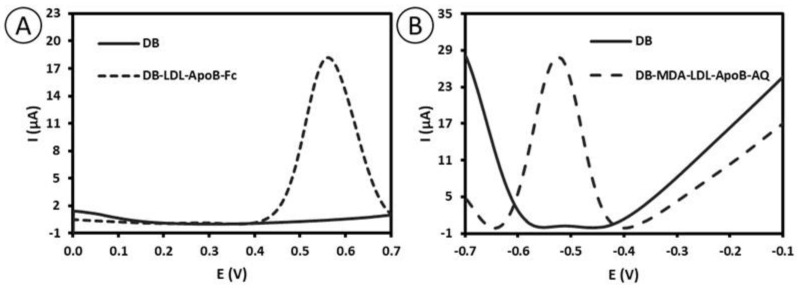
Examples of square wave voltammograms (SWVs) registered for: (**A**) dynabeads (DB) un-modified and modified with monoclonal antibody to apolipoprotein B specific for low density lipoprotein (LDL) (LDL-Apo-B) and ferrocene (Fc) (DB-LDL-ApoB-Fc) and (**B**) DB-coated monoclonal antibody to malondialdehyde (MDA) modified LDL (MDA-LDL-Apo-B) and anthraquinone (AQ) (DB-MDA-LDL-ApoB-AQ) deposited on screen-printed carbon electrodes (SPCEs) in the following buffer: 50 mM Tris, 0.15 M Na_2_SO_4_ pH 7.0. The measuring parameters of SWV: step potential = 1 mV; square wave frequency = 50 Hz; amplitude = 50 mV.

To confirm the efficient process of redox agent molecules conjugation on the surface of magnetic beads, a square wave voltammetry experiment was conducted for dynabeads un-modified and upon antibodies and ferrocene or anthraquinone deposition on beads, in buffer containing 50 mM Tris, 0.15 M Na_2_SO_4_ pH 7.0 on the carbon screen-printed electrodes. Figure 1 shows the square wave voltammograms of un-modified dynabeads (solid lines, Figure 1A,B) recorded at the positive and negative potential window. SWVs performed for antibodies and ferrocene (Figure 1A) or anthraquinone (Figure 1B)-coated dynabeads deposited on carbon screen-printed electrodes resulted in the appearance of two peaks: first, at the position of +0.544 ± 0.019 V (*n* = 3), related to the ferrocene (Figure 1A) and second, at the position of −0.531 ± 0.010 V (*n* = 4) characteristic for anthraquinone (Figure 1B). To further demonstrate the presence of surface-coated antibodies on both kinds of dynabeads, the appropriate dynabeads: DB-LDL-ApoB-Fc and DB-MDA-LDL-ApoB-AQ were incubated with their specific analytes, LDL and MDA-LDL, respectively. In addition, the non-specific effect of LDL on DB-MDA-LDL-ApoB-AQ and MDA-LDL on DB-LDL-ApoB-Fc was investigated. The decrease in current was observed, when DB-LDL-ApoB-Fc interacted with LDL and no changes were observed with MDA-LDL. Similar phenomena were observed for DB-MDA-LDL-ApoB-AQ, which proves the specificity of dynabeads modified with appropriate antibodies.

### 2.3. Quantitative Simultaneous Electrochemical Detection of LDL and MDA-LDL

The main goal of this study was to develop a platform for direct electrochemical simultaneous detection of LDL and MDA-LDL. In this case, antibody-ferrocene conjugates or antibody-anthraquinone-coated dynabeads when embedded on the surface carbon screen-printed electrodes via magnetic field underwent redox reactions. We monitored oxidation/reduction in ferrocene or anthraquinone before and after the creation of immunocomplex between monoclonal antibody to apolipoprotein B and LDL and monoclonal antibody to malondialdehyde (MDA)-modified LDL and MDA-LDL, respectively. The generation of this complex caused the spatial blocking of modified dynabeads, resulting in the decreasing of current values of respective redox agents. The changes in oxidation/reduction current were related with the concentration of analyte: LDL or MDA-LDL in the sample. They were the basis of analytical signal generation.

The first step of the proposed procedures concerned the registration of square wave voltammograms of DB-LDL-ApoB-Fc or DB-MDA-LDL-ApoB-AQ, upon immobilization on SPCEs in buffer 50 mM Tris, 0.15 M Na_2_SO_4_ pH 7.0, in the absence of specific antigens. We noticed a peak current in the range of 18.1 ± 1.4 µA, the potential of +0.544 ± 0.019 V for DB-LDL-ApoB-Fc and 28.0 ± 2.9 µA, the potential of −0.531 ± 0.010 V for DB-MDA-LDL-ApoB-AQ, derived for Fc and AQ, respectively. Next, the suitable modified beads were incubated with a solution of LDL or MDA-LDA at the particular concentrations for 30 min. Upon washing, the beads were magnetically collected on the carbon working electrode surface and, upon the addition of the measuring buffer, the SWVs were recorded. The sensing area of the carbon working electrode had a diameter of 4 mm.

**Figure 2:**
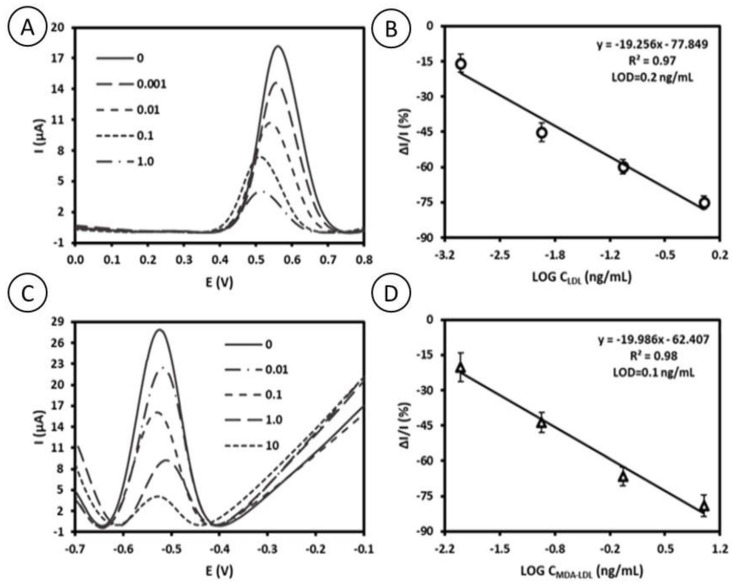
(**A**,**C**) The example of square wave voltammograms registered for immunosensing platforms at the particular concentrations of (**A**) LDL (0.001, 0.01, 0.1, 1.0 ng/mL) and (**C**) MDA-LDL (0.01, 0.1, 1.0, 10.0 ng/mL). Voltammograms were recorded until a stable reading was obtained, averaging up to 5 runs. (**B**,**D**) The dependence of the relative changes of ferrocene (**B**) and anthraquinone (**D**) redox current on the log concentration of LDL and MDA-LDL (ΔI = I_n_ − I, I_n_—peak current value obtained at particular “n” concentration of LDL or MDA-LDL, I—peak current value obtained in buffer free of antigen (*n* = 3). The measuring parameters of SWV: step potential = 1 mV; square wave frequency = 50 Hz; amplitude = 50 mV. Buffer: 50 mM Tris, 0.15 M Na_2_SO_4_ pH 7.0.

The decrease in peak current was measured, in the range from 0.001 to 1.0 ng/mL for LDL and 0.01 to 10.0 ng/mL for MDA-LDL (see Figure 2). With an increasing concentration of LDL or MDA-LDA, the number of immunocomplexes formed between DB-LDL-ApoB-Fc and LDL or DB-MDA-LDL-ApoB-AQ and MDA-LDL increased. The spatial blocking occurred as a consequence and the “local” environment of redox active agents, ferrocene and anthraquinone was changed. In effect, the decrease in signal intensity of Fc and AQ current was noticed. Similar phenomena were previously published by other authors [27,30] and by us [20].

The linear relationship between relative changes of redox currents and log concentration of LDL and MDA-LDL were proved (the regression equation of: ΔI/I = −19.256logC_LDL_ − 77.849 (R^2^ = 0.97) and ΔI/I =−19.986logC_MDA-LDL_ − 62.407 (R^2^ = 0.98)). The following formula was used to determine the detection limit (LOD):

(1) $$LOD=\frac{3.3\sigma }{s}$$

where σ means the standard deviation of the response, *s* means the slope of the calibration curve. The detection limits were determined as 0.2 ng/mL and 0.1 ng/mL for LDL and MDA-LDL, respectively. It is worth highlighting that immunosensors platforms presented offer ultrahigh sensitivity. The LODs values calculated in this work are far from elevated values of both biomarkers in the human body. Therefore, the tested sample can be diluted about 10^7^ (or 10^9^) times to obtain the detection limit estimated for LDL (or MDA-LDL). Hereby, this operation had an influence on the reduction in biological matrix effect and the possibility of interferences due to the presence of serum components.

Electrochemical magnetoimmunosensors recently became a useful tool for the determination of analytes important in the field of clinical, food and environmental analysis [31,32,33,34,35]. Most of them were constructed in sandwich configuration using the enzyme-labelled antibodies. Therefore, such configuration requires many incubation and washing steps and addition of electron transfer mediator and enzyme substrate to the solution. Under the method proposed in this paper, the interaction between antibody and antigen was evaluated by the changes in the activity of electroactive magnetic beads-coated antibodies.

The literature overview was carried out. It revealed that there was only one example of electrochemical sandwich type aptasensor for the detection of LDL based on ferrocene (Fc) and aptamer (Apt)-modified metal organic framework (MOF), magnetic silica beads (Fe_3_O_4_@SiO_2_) and disposable screen-printed electrodes (SPEs) [36]. Authors highlighted that the aptasensor could detect LDL with the detection limit of 0.3 ng/mL in plasma during 20 min and the sample volume of 0.1 mL. However, it is worth highlighting that this system was based on sandwich complex formation of Fe_3_O_4_@SiO_2_/LDL/MOFs-Fc@Apt. Thus, the additional step of complex creation and washing is required. Moreover, the metal organic frameworks nanoparticles as a matrix carrier of ferrocene are needed. Even if aptamers are used as a probe, the very similar detection limit was obtained by the method presented as compared to the system described in this work. To the best of our knowledge, the electrochemical magneto apta- or immunosensors for detection of MDA-LDL were not previously presented.

### 2.4. Repeatability, Reproducibility, Selectivity and Stability of LDL and MDA-LDA Magnetoimmunosensors

**Figure 3:**
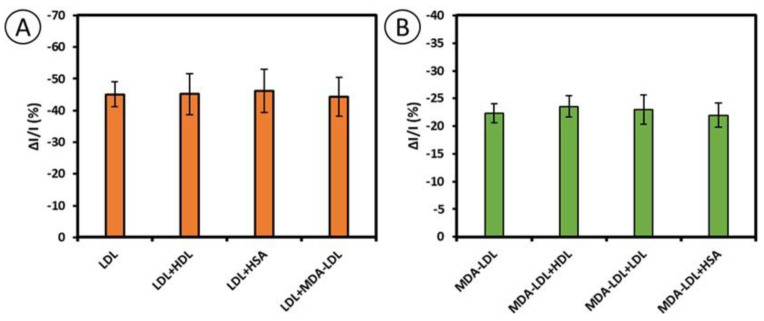
The relative changes of (**A**) ferrocene redox current registered for magnetoimmunosensor in the presence of 0.001 ng/mL of LDL along with different interferents at concentration of 0.05 ng/mL HDL, HSA and MDA-LDL and (**B**) anthraquinone redox current registered for magnetoimmunosensor in the presence of and 0.01 ng/mL of MDA-LDL along with different interferents at concentration of 0.5 ng/mL HDL, HSA and MDA-LDL.

The results of repeatability, reproducibility, selectivity and stability studies proved the practical application of platforms developed. Satisfactory repeatability of methods proposed was estimated based on relative standard deviations (RSD) about values of 7.7% for LDL and 3.8% for MDA-LDL. Additionally, sufficiently good reproducibility of both platforms were observed, characterized by RSD values of 6.0% for LDL and 7.2% for MDA-LDL detection. The selectivity studies were conducted by measuring the response of magnetoimmunosensor platforms for detection of LDL and MDA-LDL, respectively, in the presence of some potential interfering molecules: LDL and MDA-LDL vice versa, human serum albumin (HSA), high density lipoprotein (HDL). The relative changes of peak current were measured for 0.001 ng/mL LDL or 0.01 ng/mL MDA-LDL in the presence of 50-fold higher concentration of MDA-LDL, LDL, respectively, HSA and HDL. Figure 3 shows that all tested interfering proteins did not significantly affect the registered relative changes of current. Thus, it is important to highlight that electrochemical magnetoimmunosensors developed were characterized by high selectivity.

**Figure 4:**
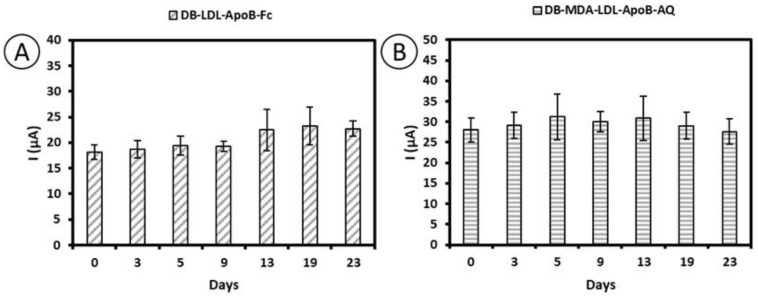
The results of stability studies estimated based on current values of (**A**) DB-LDL-ApoB-Fc and (**B**) DB-MDA-LDL-ApoB-AQ immunoconjugates measured after the preparation and next 23 days. Both immunoconjugates stored in buffer: 50 mM Tris, 0.15 M Na_2_SO_4_ pH 7.0 at 4 °C.

To determine immunocomplexes stability, the peak current of ferrocene and anthraquinone conjugates with specific antibodies and dynabeads were monitored for 23 days (Figure 4A,B). Both magnetoimmunosensors were stored in the measuring buffer of 50 mM Tris, 0.15 M Na_2_SO_4_ pH 7.0 and 4 °C. Ferrocene peak current value displayed some differences within 23 days. However, it did not exceed more than 107% of its initial value, as observed for 9 days of storing. However, anthraquinone peak values were more stable as compared with ferrocene and were below 111% of initial values during 23 days of storing. So, we can conclude that DB-LDL-ApoB-Fc immunoconjugates can be used for up to 9 days of storing, whereas DB-MDA-LDL-ApoB-AQ immunoconjugates are stable over the period of 23 days.

### 2.5. Application of Magnetoimmunosensing Platforms in Serum Sample Analysis

To determine the suitability of magnetoimmunosensing platforms for analysis in serum samples, LDL and MDA-LDL were detected in 100-fold diluted commercial human serum. The solutions of LDL and MDA-LDL were prepared in diluted serum and square wave voltammetry measurements were conducted using both platforms. As presented in Table 1, the recovery values were in the range of 100.7 to 104.0 for LDL and 100.2 to 106.0 for MDA-LDL, and relative standard deviations (RSD) of 1.9% and 3.6% for LDL and MDA-LDL, respectively. Thus, the electrochemical magnetoimmunoplatforms clearly demonstrated the possible applicability in real sample analysis.

## 3. Materials and Methods

### 3.1. Chemicals and Materials

Dynabeads functionalized with amine groups (DB-NH_2_, Dynabeads^TM^ M-270 Amine) and Apolipoprotein B Monoclonal Antibody (LDL-apoB) were obtained from Invitrogen (Poland). Malondialdehyde ApoB-100 Monoclonal Antibody (MDA-LDL-apoB) was purchased from MyBioSource, Inc. (San Diego, CA, USA). Malondialdehyde-Modified LDL (MDA-LDL) was obtained from Cell Biolabs, Inc. (San Diego, CA, USA). Ferrocene Carboxylic N-hydroxysuccinimide Ester (Fc-NHS) was acquired from Fivephoton Biochemicals (San Diego, CA, USA). N-Hydroxysuccinimide (NHS), N-(3-Dimethylaminopropyl)-N-ethylcarbodiimide hydrochloride (EDC), Anthraquinone-2-carboxylic acid (AQ-COOH), Na_2_SO_4_, trizma base (Tris), 2-(N-Morpholino)ethanesulfonic acid hydrate (MES), dimethyl sulfoxide (DMSO), low density lipoprotein (LDL) and humans serum albumin (HSA) were purchased from Sigma-Aldrich (Poznań, Poland). High density lipoprotein (HDL) was bought from EMD Millipore Corporation (Burlington, MA, USA). Ethanol and methanol were purchased from POCH (Gliwice, Poland). Deionized water (resistivity of 18.2 MΩ cm; Millipore Mili-Q, Bedford, MA, USA) was used for preparation of all aqueous solutions. Human blood serum was obtained from Sigma-Aldrich.

### 3.2. Apparatus and Electrodes

Potentiostat/Galvanostat AutoLab (Methrohm, Herisau, The Netherlands) was used for all electrochemical measurements. The screen-printed carbon electrodes (SPCEs, DRP-150), cable (CAC), boxed connector (DSC), magnetic support for screen-printed electrodes were purchased from Methrom Hispania, S.I., (Madrid, Spain). The SPCE consisted of: carbon working electrode (4 mm diameter), platinum auxiliary electrode and silver reference electrode. Magnetic Separation Stands (Promega Corporation, Fitchburg, WI, USA) was used to efficiently separate and handle dynabeads.

### 3.3. Preparation of Magnetoimunoconjugates for LDL and MDA-LDL Detection

Prior to modification, 0.1 mg/mL LDL-ApoB antibodies were activated for 30 min in a mixture solution containing of EDC/NHS (20 mM each) prepared in H_2_O. The 3 µL aliquot of the DB-NH_2_ solution was transferred into a 1.5 mL centrifuge tube and washed twice with 50 µL of 0.025 M MES buffer, pH 5.0. At each washing step, the beads were re-suspended and then, the supernatant was discarded by magnetic separation. Additionally, in order not to allow the beads to aggregate before each washing step, the tube with the mixture was sonicated for 1 s. Then, the DB-NH_2_ was incubated with 25 µL solution of 0.1 mg/mL antibodies LDL-ApoB and 25 µL solution of 1 mg/mL Fc-NHS in DMSO/H_2_O mixture for 60 min (RT, gently shaking). The resulting DB-LDL-ApoB-Fc was washed twice: first with 50 µL of 0.025 M MES buffer, pH 5.0 and then, with 50 µL of 50 mM Tris, 0.15 M Na_2_SO_4_ pH 7.0. The DBs bearing the capture LDL-ApoB and redox-tag Fc were subsequently used for LDL determination.

The DB-MDA-LDL-ApoB-AQ immunoconjugates were prepared similarly to the procedure described above. With the difference that, the carboxylic acid groups from AQ were activated by dissolving in a solution of EDC/NHS (20 mM each) in a mixture of DMSO/H_2_O and were gently mixed for 30 min, at RT and malondialdehyde ApoB-100 monoclonal antibody were used.

### 3.4. Electrochemical Determination of LDL and MDA-LDL

The determination of LDL or MDA-LDL involved their incubation with DB-LDL-ApoB-Fc, DB-MDA-LDL-ApoB-AQ, respectively. The 25 µL of a mixture solution containing the appropriate concentration of standard LDL or MDA-LDL (prepared in 50 mM Tris, 0.15 M Na_2_SO_4_ pH 7.0) or supplemented with 100 diluted serum sample were added to suitable immunoplatform for 30 min (RT, gently shaking). Afterwards, the DB-LDL-ApoB-Fc-LDL and DB-MDA-LDL-ApoB-AQ-MDA-LDL were washed twice and then, re-suspended in 50 µL of 50 mM Tris, 0.15 M Na_2_SO_4_ pH 7.0 to carry out the electrochemical determination at SPCE. The fabrication of the DBs redox active immunoplatforms is depicted in the Scheme 1. Prior to measurement, the SPCEs were rinsed with water and ethanol. Then, they were sonicated for 2 min in ethanol. Afterwards, they were rinsed with water and allowed to dry. To carry out the electrochemical measurement, the prepared magnetoimmunoconjugates suspension were deposited onto the surface of the clean SPECs working electrodes. The magnetic support was placed under the working electrode to collect the DBs immunoconjugates onto it. The 50 μL of the DB-LDL-ApoB-Fc suspension was deposited onto the surface of the SPEC working electrode. Then, the 50 μL of 50 mM Tris, 0.15 M Na_2_SO_4_ pH 7.0 was additionally added. Afterwards, the electrochemical measurement was carried out. Finally, the electrochemical signals (square wave voltammograms) of the immunosensor was recorded and LDL were detected based on the changes of redox response signals of ferrocene as the electroactive agent. The same procedure was performed for detection MDA-LDL by DB-MDA-LDL-ApoB-AQ-MDA-LDL biosensor.

## 4. Conclusions

In this work, we reported an immunosensing platforms involving the use of: (1) magnetic dynabeads (DBs) conjugated with specific antibodies and redox active agents and (2) screen-printed carbon electrodes (SPCEs) for simple and relatively fast simultaneous electrochemical determination of LDL and MDA-LDL. Two types of redox active magnetoimmunoconjugates were prepared consisting of: (1) apolipoprotein B monoclonal antibody (LDL-apoB) and ferrocene (Fc) (DB-LDL-apoB-Fc) and (2) malondialdehyde ApoB-100 monoclonal antibody (MDA-LDL-apoB) and anthraquinone (AQ) (DB-MDA-LDL-apoB-AQ). Upon the incubation of appropriate immunoconjugates with specific antigens, and following their magnetic capturing on the surface of SPCEs, the voltammetry signals originating from redox active agents were registered. The total determination time did not exceed 45 min, including the interaction time between beads and analytes and electrochemical measurement. The changes in the redox current of electroactive magnetoimmunoconjugates measured before and after contact with specific antigens were the basis of their determination. The immunoplatforms prepared showed high sensitivity characterized by limit of detection in ng/mL level for LDL (0.2 ng/mL) and MDA-LDL (0.1 ng/mL), sufficient selectivity towards possible interferents, as HDL and HSA, and relatively good stability.

## Figures and Tables

**Table 1:** Results of LDL and MDA-LDL detection in 100-folded human serum samples.

Spiked C_LDL_ (ng/mL)	Measured C_LDL_ (ng/mL)	Recovery (%)	RSD (%)	Spiked C_MDA_-_LDL_ (ng/mL)	Measured C_MDA-LDL_ (ng/mL)	Recovery (%)	RSD (%)
0.2	0.208	104.0	1.9	0.1	0.106	106.0	3.6
0.5	0.503	101.2	1.6	0.5	0.505	101.4	2.1
1.0	1.007	100.7	0.2	1.0	1.002	100.2	1.1

## Data Availability

The data presented in this study are available on request from the corresponding author.
